# Impact of family childhood adversity on risk of violence and involvement with police in adolescence: findings from the UK Millennium Cohort Study

**DOI:** 10.1136/jech-2024-223168

**Published:** 2025-01-21

**Authors:** Nicholas Kofi Adjei, Kenisha Russell Jonsson, Jones Opoku-Ware, Sanni Yaya, Yanhua Chen, Davara Bennett, Ruth McGovern, Luke Munford, Michelle Black, David Taylor-Robinson

**Affiliations:** 1 Department of Public Health, Policy and Systems, University of Liverpool, Liverpool, UK; 2 School of Public Health and Community Medicine, Institute of Medicine, Gothenburg University, Göteborg, Sweden; 3 Department of Sociology and Social Work, Kwame Nkrumah University of Science and Technology, Kumasi, Ghana; 4 University of Parkou, Parakou, Benin; 5 Population Health Sciences Institute, Newcastle University, Newcastle, UK; 6 Manchester Centre for Health Economics, University of Manchester, Manchester, UK

**Keywords:** POVERTY, ADOLESCENT, COHORT STUDIES, CLUSTER ANALYSIS

## Abstract

**Background:**

Childhood adversities, such as exposure to parental mental illness, domestic violence and abuse, substance use, and family poverty, have been linked to involvement in violence in early adulthood. However, evidence on the cumulative impact of multiple adversities throughout childhood on violence and crime in adolescence remains scarce. This study investigates the associations between trajectories of family adversity and poverty during childhood, and the risk of involvement in violence and contact with police in adolescence.

**Methods:**

We used longitudinal data from the UK Millennium Cohort Study on 9316 children. Exposure trajectories of family adversities and poverty were characterised (from ages 0–14 years) using group-based multi-trajectory models. The outcomes were weapon involvement, for example, carrying a knife, and police contact measured at age 17 years. Odds ratios and 95% confidence intervals (OR, 95% CI) and population attributable fractions were estimated using logistic regression models, adjusting for confounding factors.

**Results:**

The prevalence of weapon involvement and contact with police at age 17 years were 6.1% and 20.0%, respectively. Compared with children who experienced low poverty and family adversity throughout childhood, those exposed to persistent poverty and poor parental mental health were at notably increased risk of carrying weapons (adjusted OR (aOR) 2.2, 95% CI 1.3 to 3.6) and reporting contact with police (aOR 2.1, 95% CI 1.6 to 2.8). We estimate that about 32% of weapon involvement and 23% of contact with police at age 17 were attributable to persistent poverty and family adversity.

**Conclusion:**

Exposure to poverty and poor parental mental health throughout childhood doubles the risk of weapon involvement and police contact in early adulthood. These findings emphasise the importance of lifecourse and anti-poverty approaches to reducing involvement in crime in the UK.

**WHAT IS ALREADY KNOWN ON THIS TOPIC**

- Adverse childhood experiences are linked to an increased risk of involvement in violence during early adulthood.

**WHAT THIS STUDY ADDS**

- Using longitudinal data from a contemporary and representative cohort of UK children, this study found that exposure to poverty and family adversity either singly or in combination throughout childhood significantly increases the likelihood of violence and criminal justice involvement during adolescence.
- An estimated 32% of weapon involvement and 23% of police contact at age 17 were attributable to persistent poverty and family adversity.

**HOW THIS STUDY MIGHT AFFECT RESEARCH, PRACTICE OR POLICY**

- Addressing child poverty and family adversity comprehensively and syndemically earlier in the life course across multiple sectors may reduce risk-taking behaviours in adolescence, ultimately contributing to better outcomes for young people.

## Introduction

 Childhood family adversities, including exposure to parental mental illness, domestic violence and abuse, substance use, and poverty, are known to co-occur or cluster together.^1 2^ The cumulative impact of these adversities has been linked to trauma, mental ill-health, and socio-emotional behavioural problems in adolescence.^2 3^ Research has also shown that as children grow, the impact of early-life adversities may become more pronounced in later years.^4^ These lasting effects may result not only in persistent physical and mental health issues^2 3^ but also in a heightened risk of violence and involvement with the criminal justice system.^5 6^

Youth crime and violence are common issues globally. While the prevalence may vary across regions, they are still a significant concern in many countries.^7 8^ In England and Wales, for example, a recent report indicated that approximately 104 400 first-time entrants were recorded in the criminal justice system in 2020, with 11% of these entrants being children and adolescents aged 10 to 17 years.^8^ Among these offences, ‘violence against the person’—encompassing minor offences such as harassment and common assault, as well as serious offences like murder, actual bodily harm, and grievous bodily harm—remains prevalent (31%). Preventing youth crime and violence is therefore a significant public health priority due to the long-term personal, social, and economic consequences, including incarceration, poor mental and physical health, social stigma, and disrupted life trajectories, which contribute to adverse outcomes later in life.^9^

Several risk factors, including family socioeconomic circumstances, psychosocial risks and environmental influences, have been linked to adolescent risk-taking and involvement in violence.^10 11^ However, a probable and often overlooked risk factor is childhood family adversity, particularly its cumulative effect over a longer period of time. Although some studies have investigated the relationship between adverse childhood experiences and adolescent violent offending,^12^ as well as contact with the criminal justice system,^5^ the complex interrelationships between multiple family-related risk factors across the early life course still remain unclear. Since risk factors tend to cluster^13^ and may accumulate over the life course,^14^ developing appropriate interventions and preventative strategies requires an assessment of multiple risk factors across different developmental periods, and using multiple trajectories to characterise family adversity enables us to capture the complexity and timing of these risks.^15^ Therefore, the current study builds on our previous work on the clustering of family adversities across the early life course,^2^ to assess the extent to which involvement in youth violence, crime and contact with the justice system may be predicted by early life trajectories of poverty and family adversities. We further quantified the contribution of exposure to family adversity and poverty trajectories to the risk of youth crime and violence at the country level.

## Methods

### Study setting and participants

We used data from the Millennium Cohort Study (MCS), a nationally representative UK population-based cohort study. The MCS tracks the lives of over 18 000 children born between September 2000 and January 2002, following them over time at ages 9 months (wave 1), 3 years (wave 2), 5 (wave 3), 7 (wave 4), 11 (wave 5), 14 (wave 6), and 17 years (wave 7). The respective numbers of responding families at each wave were 18 552, 15 590, 15 246, 13 857, 13 287, 11 726, and 10 625. At each wave, information on a variety of topics, including socioeconomic circumstances, family structure, health, and cognitive development was collected from the main caregiver, usually the child’s mother. At age 17, parental involvement in the survey was minimal; cohort members provided information on a wide variety of domains, including relationship with parents and risky behaviours. Detailed information on the survey design, sampling, and scope of MCS is provided elsewhere.^16^

### Exposures

Six trajectory groups of poverty and family adversities (ie, parental mental illness, domestic violence, and alcohol use) were identified in our previous study^2^ and used as exposures in this current study. A group-based multi-trajectory modelling technique was used to derive these six trajectory groups.^17^ The ‘low poverty and adversity’ trajectory group (43.2%) comprises children with low exposure to childhood poverty and family adversities over time. The ‘persistent poverty’ trajectory group (22.6%) includes children with a high likelihood of experiencing continuous poverty throughout their childhood. The ‘persistent poor parental mental health’ trajectory group (11.9%) is characterised by consistently high rates of poor parental mental health. The ‘persistent parental alcohol use’ (7.7%) and ‘persistent domestic violence’ (34%) trajectory groups consist of children continually exposed to parental alcohol use and domestic violence, respectively. Lastly, the ‘persistent poverty and poor parental mental health’ trajectory group (11.1%) comprises children with high exposure to the combination of both persistent poverty and poor parental mental health throughout their childhood (online supplemental appendix 1). Description of measurements assessed for trajectory exposures can be found in online supplemental appendix 2.

### Outcomes

Two outcomes at age 17 years were used in the analysis: weapon involvement, and police contact. Weapon involvement was assessed by asking participants two questions: whether in the last 12 months they had ‘carried a knife or other weapon for protection, because someone asked them to, or in case it was needed in a fight’, and whether in the past year they had ‘hit someone with or used a weapon’. Participants who answered ‘yes’ to either question were identified as having been involved in weapon use. To assess police contact, we used questions on whether participants had ever been: (1) stopped and questioned by the police; (2) given a formal warning or caution by a police officer; and (3) arrested by a police officer and taken to a police station. Participants who reported any form of police contact were assigned a value of 1, whereas those reporting no police contact were assigned a value of 0. We further examined all the three questions separately in secondary analyses.

### Confounders

Potential confounders were selected based on previous research, guided by a directed acyclic graph (online supplemental appendix 3). These included child’s sex, maternal ethnicity (white, mixed, Indian, Pakistani and Bangladeshi, black or black British, or other ethnic groups), and maternal education (degree or higher, diploma, A-levels, GCSE A–C, GCSE D–G, or none) when the child was aged 9 months.

### Statistical analysis

First, we characterised the exposure trajectories of poverty and family adversities from the age of 9 months to 14 years using our previously developed group-based, multi-trajectory models with the Traj procedure in Stata, version 16.0 (Stata Corp, College Station, TX, USA).^17^ Second, percentages were used to illustrate the prevalence of weapon involvement and police contact. Differences in prevalence were examined using Pearson’s χ² test. Third, we assessed the association between the six identified trajectory groups and the outcomes using logistic regression models with 95% confidence intervals (95% CI). Two models were built: model 1 is the crude model, model 2 is the adjusted model. Both models included longitudinal weights to account for response bias, attrition, and sampling design. All analyses were conducted using multiple imputed data (chained equations, 25 imputations) to address missingness in the outcome (<30% of observations) and the explanatory variables (<10% of observations). Fourth, we estimated population-attributable fractions (PAFs)^18^ to assess the proportion of weapon involvement and contact with police that could be prevented if exposure to poverty and family adversity were eliminated or reduced to the levels of children who experience low poverty and adversity (see online supplemental appendix 4 for more details on the model specification). The statistical analyses were carried out using Stata (version 16.0).

## Results

### Study population characteristics

**Figure 1:**
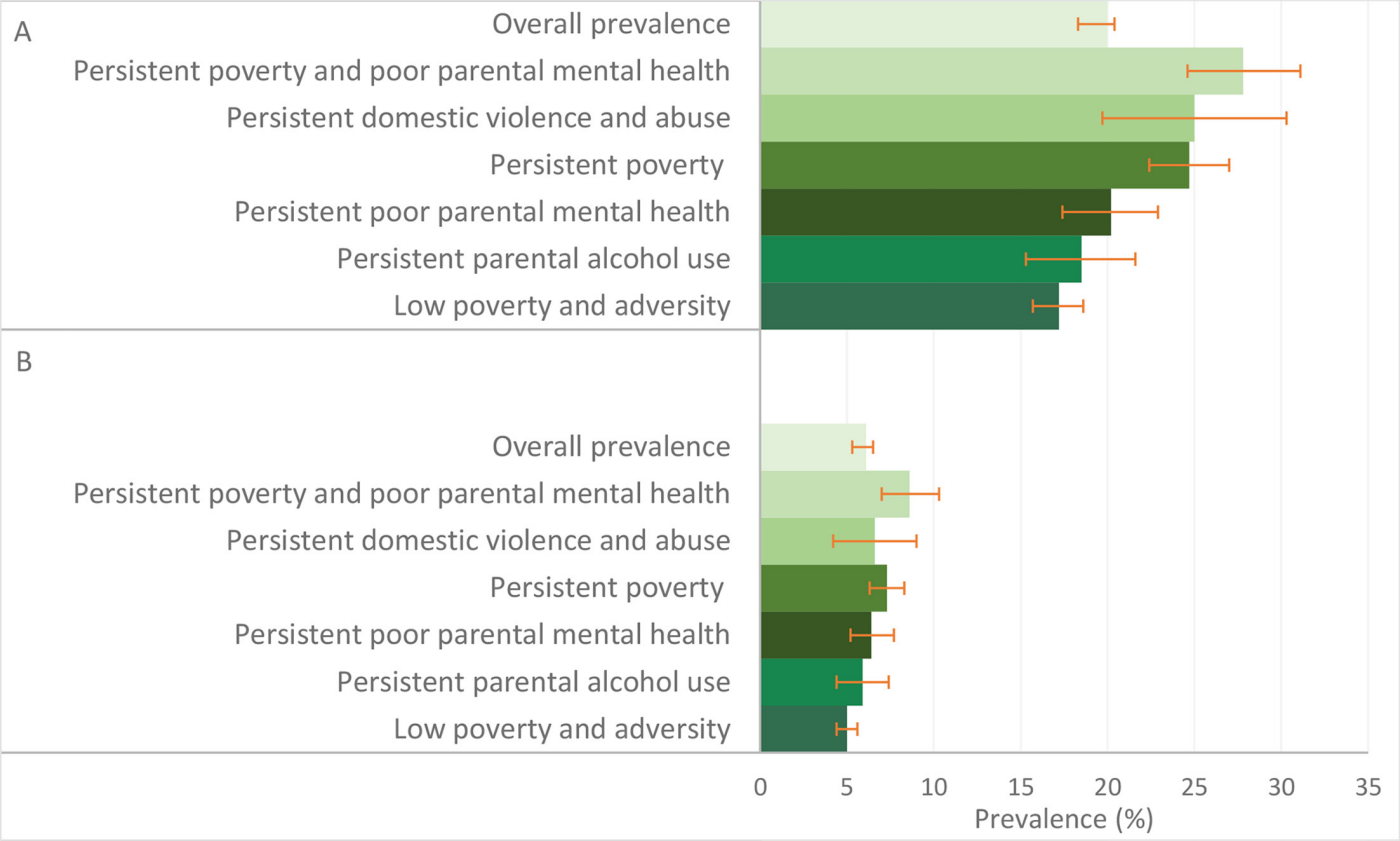
Prevalence of police contact (panel A) and weapon involvement (panel B) by poverty and family adversity trajectories in the UK Millennium Cohort Study at age 17 years.

Of the 14 443 families who were eligible at age 17 (wave 7), 9316 families were analysed (online supplemental appendix 5). At age 17, the overall prevalence of weapon involvement and contact with police were 6.1% and 20.0%, respectively (table 1). The prevalence of weapon involvement and contact with police by the six poverty and adversity trajectory groups is shown in figure 1. Children exposed to poverty and family adversity in early years were more likely to use a weapon and have contact with police in adolescence. For example, the prevalence of weapon involvement was 8.6%, and contact with police was 27.8% for children in the persistent poverty and poor parental mental health trajectory group, compared with 5.0% and 17.2%, respectively, for children in the low adversity and poverty trajectory group (figure 1).

**Table 1:** Overall prevalence of weapon involvement and contact with police in the UK Millennium Cohort Study at age 17 years (N=9316)

Characteristics	Weapon involvement	Contact with police
Overall prevalence	6.1%	20.0%
Child’s sex		
Boy	78.7%	63.2%
Girl	21.3%	36.8%
Maternal education		
Degree plus	15.7%	17.2%
Diploma	8.0%	9.0%
A-levels	8.3%	8.7%
GCSE A–C	32.0%	32.1%
GCSE D–G	11.2%	11.9%
None	24.8%	21.1%
Maternal ethnicity		
White	83.6%	88.4%
Mixed	0.7%	1.1%
Indian	2.6%	2.1%
Pakistani and Bangladeshi	9.3%	5.1%
Black or black British	2.0%	2.3%
Other ethnic groups	1.8%	1.0%
Trajectories of poverty and family adversity		
Low poverty and adversity	32.2%	37.6%
Persistent parental alcohol use	6.4%	7.7%
Persistent domestic violence and abuse	5.5%	4.5%
Persistent poor parental mental health	13.6%	11.9%
Persistent poverty	25.7%	24.5%
Persistent poverty and poor parental mental health	16.6%	13.7%

GCSE, General Certificate of Secondary Education.

### Associations of poverty and family adversity trajectories with weapon involvement and contact with police

**Figure 2:**
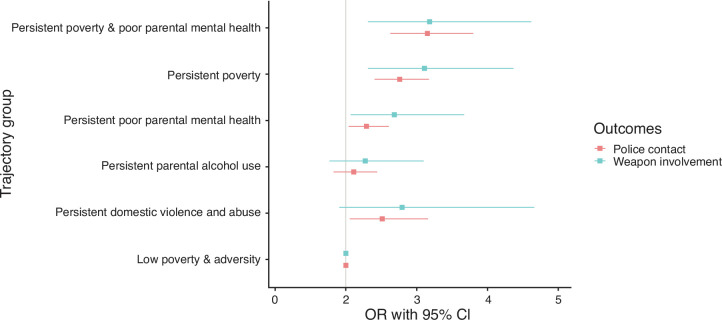
Associations of predicted poverty and family adversity trajectories with police contact and weapon involvement at age 17 years in the UK Millennium Cohort Study. Models adjusted for child’s sex, maternal education, and maternal ethnicity.

Figure 2 shows the associations between identified trajectory groups, weapon involvement and contact with police at age 17. After adjusting for confounders, children in the persistent family adversity trajectory groups remained at increased risk of carrying weapons and reporting any form of police contact compared with those who experienced low poverty and adversity throughout childhood. The associations were particularly strong for children exposed to both persistent poverty and poor parental mental health. For example, compared with children exposed to low poverty and adversity throughout childhood, those who experienced persistent poverty and poor parental mental health had higher odds of carrying weapons (adjusted OR (aOR) 2.2, 95% CI 1.3 to 3.6) and reporting contact with police (aOR 2.1, 95% CI 1.6 to 2.8). The adjustment for covariates did not result in any substantial attenuation of the results when compared with the crude model (see online supplemental appendix 6).

**Figure 3:**
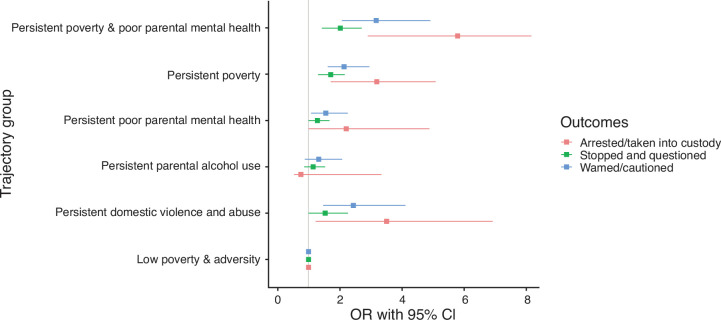
Associations of predicted poverty and family adversity trajectories and police contact at age 17 years in the UK Millennium Cohort Study. Models adjusted for child’s sex, maternal education, and maternal ethnicity.

Similar associations were observed for the various forms of police contact (figure 3). Persistent childhood adversity groups remained associated with all forms of police contact in adolescence. For example, children who experienced both persistent poverty and poor parental mental health were more than five times more likely to be arrested or taken into police custody (aOR 5.8, 95% CI 2.8 to 8.2), three times more likely to be warned or cautioned by police (aOR 3.2, 95% CI 2.1 to 4.9), and twice as likely to be stopped and questioned by police (aOR 2.0, 95% CI 1.4 to 2.7) compared with children exposed to low poverty and adversity throughout childhood.

### Population attributable fraction

**Figure 4:**
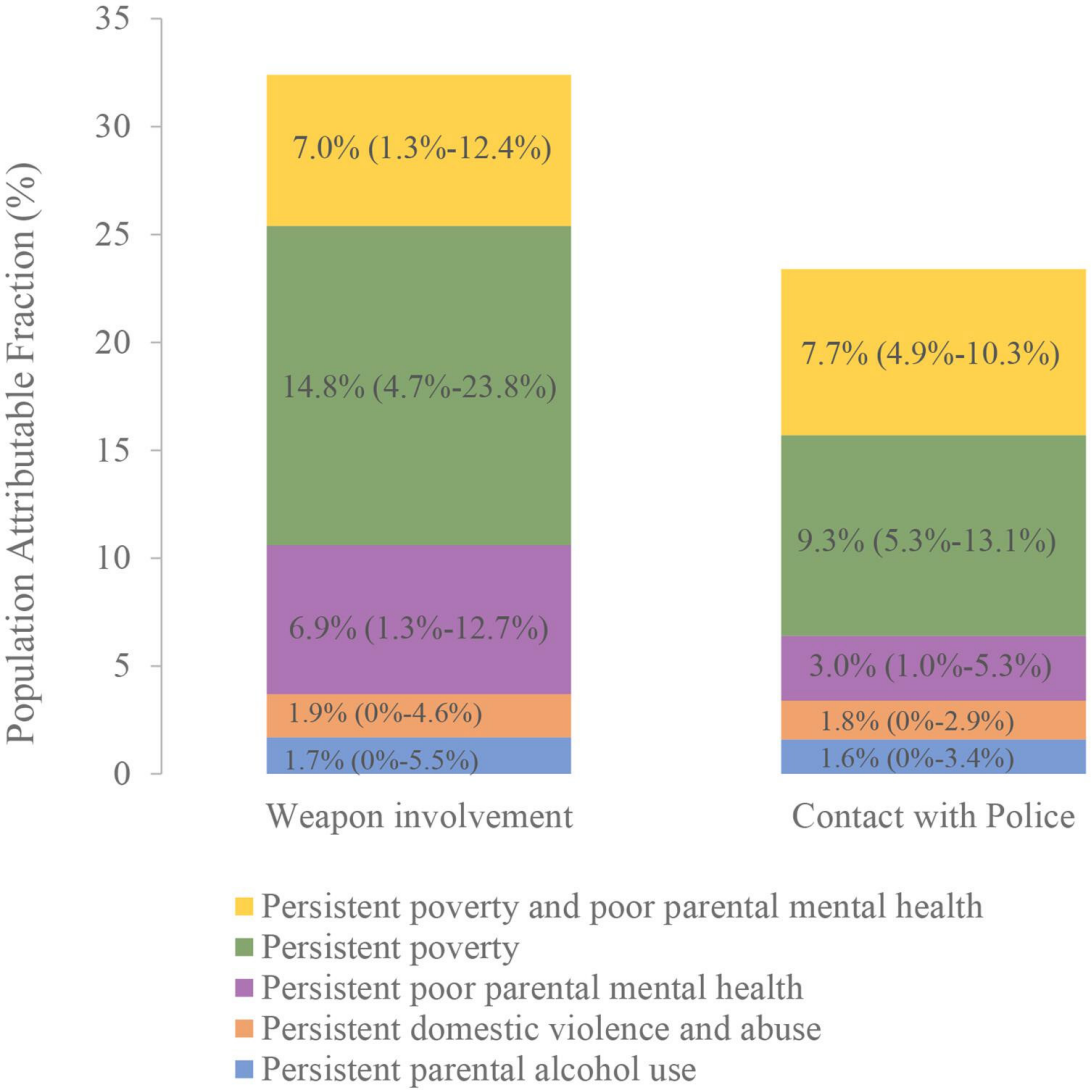
Population-attributable fractions of the trajectory groups. Compared with the low poverty and family adversity trajectory groups, the overall proportion of weapon involvement and contact with police attributable to persistent poverty and family adversity was 32.3% (95% CI 14.9% to 46.1%) and 23.4% (95% CI 16.4% to 29.7%), respectively.

The PAF estimates show the population burden of weapon involvement and police contact attributable to each trajectory group (figure 4). About 32% of the cases of weapon involvement and 24% of police contact at age 17 were attributable to persistent poverty and family adversity throughout childhood. In other words, if all children in the UK had the exposure trajectory of low poverty and family adversity, we would see a 32% reduction in weapon involvement and a 24% reduction in police contact, assuming causality. When broken down by individual trajectory, exposure to persistent poverty contributed most to the population burden, explaining about half of the additional burden due to family adversity.

## Discussion

In this large contemporary UK birth cohort, we found that about one in 16 (6%) of young people reported carrying or using weapons at age 17, and about one in five (20%) of young people reported any form of police contact. Overall, we found that children who were exposed to some degree of poverty and family adversity, either singly or in combination, throughout childhood were at increased risk of violence and criminal justice involvement in adolescence. Around one in 10 children experienced persistent risk of poverty and poor parental mental health up to age 14 which was associated with double the odds of involvement with violence and the police at age 17. We further estimate that about 32% of weapon involvement and 23% of the population burden of contact with police at age 17 were attributable to persistent poverty and family adversity throughout childhood.

The prevalence of weapon involvement and contact with the police during adolescence is consistent with the literature.^5 10^ Nonetheless, this study is one of the first to explore the long-term impact of poverty and multiple family-related risk factors, including parental mental health, alcohol use, and domestic violence and abuse, on the risk of youth crime and violence. In a recent study in the UK, Wadman and colleagues^19^ used latent class analysis to assess the impact of early familial adversity, including marital instability on adolescent risk behaviours. While their study showed that transitioning to a higher adversity group within the first 5 years of a child’s life was associated with risk-taking behaviour and criminality at age 14, the longitudinal experience of children over a longer period of time, into early adulthood, had yet to be captured. Thus, our study contributes to the limited longitudinal evidence, using a life course perspective^20^ to examine trajectories and patterns of children’s family risk exposure over the early life course and their impacts on risk-taking behaviour in adolescence. Our analysis provides strong evidence that persistent adversity throughout childhood is strongly associated with risk of involvement in violence and contact with police in adolescence, highlighting the negative effects of both accumulation and duration of childhood adversity.^14^ It is noteworthy that we did not find any statistically significant associations between the persistent parental alcohol use and domestic violence trajectory groups and police contact. This may be because the pathways linking these risk factors to police contact may be mediated by additional factors, such as socioeconomic circumstances, which could modify their impacts.^21^

The mechanisms underlying childhood adversities and adverse outcomes in later life are complex and not fully understood, but they can largely be attributed to familial–environment interactions and psychosocial mechanisms.^22 23^ Theories such as Bronfenbrenner’s ecological systems theory^24^ may thus serve as a useful framework for understanding our study results. This theory posits that a child’s development is influenced by a series of interconnected factors, ranging from immediate surroundings (eg, family) to broader societal structures. Our longitudinal studies lend some support for this theory, as we have shown that multiple factors, including family socioeconomic conditions and psychosocial family risks, co-occur or cluster together to have a strong impact on adolescent offending and crime. A number of mechanisms may explain this interaction. The level and nature of emotional support a parent is able to provide to their child is influenced by poverty and parental mental health problems.^25 26^ This challenging context, characterised by financial worry and accumulative adversity, increases the likelihood of harsh and disciplinary parenting practices.^27^ Such parenting practices have been found to mediate adolescent involvement in violent behaviour through pushing adolescents away from the family home and increasing their exposure to violent and antisocial behaviours often found in high poverty neighbourhoods.^28^

We found that 10% of children experienced persistent family poverty and persistent poor parental mental health up to the age of 14, and this co-occurrence was associated with a twofold increased risk for both weapon carrying or use and contact with the police at age 17. These findings suggest that interventions to address violence and criminal justice system involvement should not only target immediate family problems but also broader social determinants such as poverty and mental health services. A number of systematic reviews have examined the effectiveness of interventions to prevent (re)offending behaviour in adolescents,^29 30^ including knife crime and other violent behaviour,^31 32^ and show mixed results. Early selective interventions for a subpopulation of children and young people who have experienced adversity, which combines risk reduction and resilience enhancing approaches, show promise at reducing violent and other offending behaviour.^25^ These interventions should target both children and their families, providing poverty-informed integrated interventions which combine therapeutic interventions for the parent, parent skills training and support for the adolescent.^3 33^ Further, policies to redistribute income and reduce poverty^2 34^ are likely to contribute to the reduction of youth violent and offending behaviour.

A major strength of this study is the use of large, nationally representative longitudinal cohort data and repeated measures of poverty and family adversity (ie, parental mental illness, domestic violence, and alcohol use) throughout childhood and early adolescence. Nonetheless, some potential limitations exist. First, attrition and missing data are ubiquitous problems in longitudinal studies, leading to potential selection bias. However, we used multiple imputations to address missing data. We also conducted a sensitivity analysis to compare the main analysis (imputed data) to the alternative approach, complete case analysis, and found that the results did not substantively change (see online supplemental appendix 6). Second, although the associations between childhood adversity and youth violence remained after controlling for potential confounders, including ethnicity, we acknowledge that some environmental factors could confound the complex relationship under investigation. Hence, we assessed the robustness of unmeasured confounding using the E-values approach^35^ and found that the results are robust to omitted confounding (online supplemental appendix 7 and 8). In addition, the interpretation of PAFs assumes that the relationship between exposure and outcome represents a causal effect. Consequently, they should be interpreted with caution. Despite the limitations, this longitudinal study makes a significant contribution to the literature by demonstrating that accumulation of poverty and family adversity throughout childhood are associated with weapon involvement and various forms of police contact in adolescence.

In terms of policy and practice, our study shows that exposure to multiple adversities during the early developmental periods is strongly associated with risk-taking behaviours in adolescence, having implications for youth services and early intervention. The findings highlight the need for comprehensive and holistic interventions that address various aspects of children’s lives, including their family environment. Indeed, youth crime and violence are significant concerns for the UK government.^36 37^ To combat the rising rate of weapon use including knife crime among young people, the government enacted the Offensive Weapons Act 2019,^36^ introducing new offences related to a wide range of weapons and prohibiting their possession in private. Additionally, to tackle the ‘root causes’ of violence, several Violence Prevention Units have been established, bringing together multiple organisations across local communities to address risk factors through evidence-based early intervention and prevention.^36^ Despite these efforts and investments, latest statistics from the Office for National Statistics in the UK shows that knife crime increased by 7% from the year ending December 2022 to December 2023.^37^

According to the most recent population estimates, there were around 1 567 000 16- to 17-year-olds in the UK in 2023.^38^ The figures estimated here suggest that 20% of these, around 313 400 16- to 17-year-olds, will have had contact with the police. Applying costs tariffs from HM Treasury’s Green Book^39^ indicate that the annual cost of this is around £28 832 800. Analysis here shows that if all children in the UK had the exposure trajectory of low poverty and family adversity, we would see a reduction in contact with police by 23.4% (95% CI 11.2% to 35.0%). These estimates suggest that the annual saving to police expenditure would be around £6 746 880 (95% CI £3 229 280 to £10 091 480). In fact, the actual costs savings are likely to be much larger, as this is the cost of contact with police and does not account for the fact that some instances will result in further contacts and potential entry into the criminal justice system. HM Treasury’s Green Book^39^ estimates the average cost of a first time entrant (under 18) to the Criminal Justice System, in the first year following the offence, to be £4152 (2021/22 prices).

In conclusion, our findings suggest the need for a whole-system approach and the implementation and strengthening of national and local policies focused on early intervention and support for families with low incomes and those experiencing family adversity, such as mental health problems. Addressing these issues comprehensively and syndemically earlier in the life course across multiple sectors, such as schools, communities, healthcare providers, and the law system,^40^ may reduce risk-taking behaviours in adolescence, ultimately contributing to better outcomes for young people.

## Supplementary material

10.1136/jech-2024-223168 online supplemental appendix 1

## Data Availability

Data are available upon reasonable request.
