# Supplementary material for: Impact of family childhood adversity on risk of violence and involvement with police in adolescence: findings from the UK Millennium Cohort Study
Source: J Epidemiol Community Health. 2025 Jan 21;79(6):e223168. doi: 10.1136/jech-2024-223168 (PMC12171507; doi:10.1136/jech-2024-223168)
Supplement: online supplemental appendix 1 [file jech-79-6-s001.pdf]

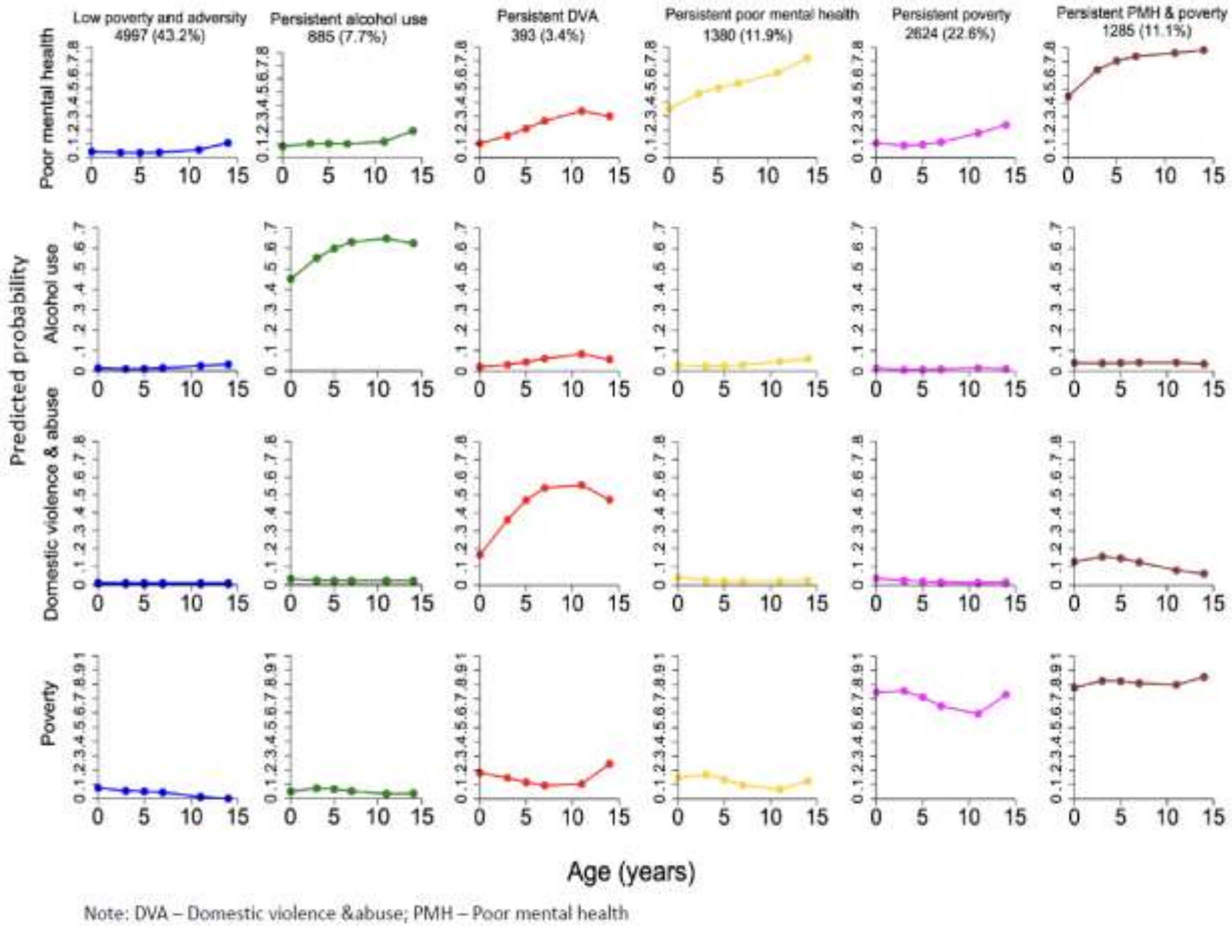

Supplementary Figure 1. Estimated trajectory groups of family adversity and poverty in the UK Millennium Cohort Study. Excerpted from Adjei et al. 2022. *The Lancet Regional Health-Europe.*[1]

Supplementary Box 1. Description of measurements assessed for trajectory exposures

- **Parental mental ill health (Child aged 9 months)** – Rutter Malaise Inventory (RMI)[16] scale was used to assess parental mental ill health· A shortened 9-item self-completed version of the RMI measuring depression, anxiety and psychosomatic illness was used· The 9-item short form included items 'feel tired most of the time', 'feel miserable or depressed', 'worried about things', 'often get into violent rage' 'suddenly become scared for no good reason', 'easily upset or irritated', 'constantly keyed up or jittery', 'every little thing gets on nerves and wears you out', and 'heart race like mad'· Scores from these items were summed, and we used a validated cut off for mental ill health ['yes (scores >=4)/no']·

- **Parental mental ill health (Child aged 3 to 14 years) –** Kessler 6 (K6)[17] scale was used to assess parental mental ill health in the last 30 days asking the responders how often they felt depressed, hopeless, restless or fidgety, worthless, or that everything was an effort· Respondents answered on a five-point scale from 1(all the time) to 5 (none of the time)· We reversed and rescaled all items from 0 to 4 for analysis purposes, so that high scores indicate high levels of psychological distress· We used a validated cutoff widely used in previous studies ['yes (scores >=6)/no']

- **Frequent parental alcohol use (Child aged 9 months to 7 years) –** the main responder answered a question about their usual frequency of alcohol consumption ('*Every day, 5-6 times per week, 3-4 times per week, 1-2 per week, 1-2 per month, less than once a month or never'*)·
  Dichotomised: [every day and 5-6 times per week (Yes) vs· 3-4 per week/1-2 per week/ 1-2 per month/never (No)]

- **Frequent parental alcohol use (Child aged 11 to 14 years) –** the main responder answered a question about the usual frequency of alcohol consumption *('>=4 times per week, 2-3 times per week, 2-4 times per month, monthly or less, or never')·*
  Dichotomised: [4 or more times a week (Yes) vs· 2-3 per week/2-4 per month/ monthly or less/never (No)]

- **Domestic violence and abuse (Child aged 9 months to 14 years) –** the main responder was asked about the use of physical force by the partner in relationship ('*Yes, No'*)

- **Poverty (Child aged 9 months to 14 years)** – relative income poverty[4], defined as household equivalised income of less than 60% of national median household income equivalised according to the Organisation for Economic Co-operation and Development (OECD) household equivalence scale

Note: References to "parental" such as parental mental ill health refers to the main carer, usually the mother.

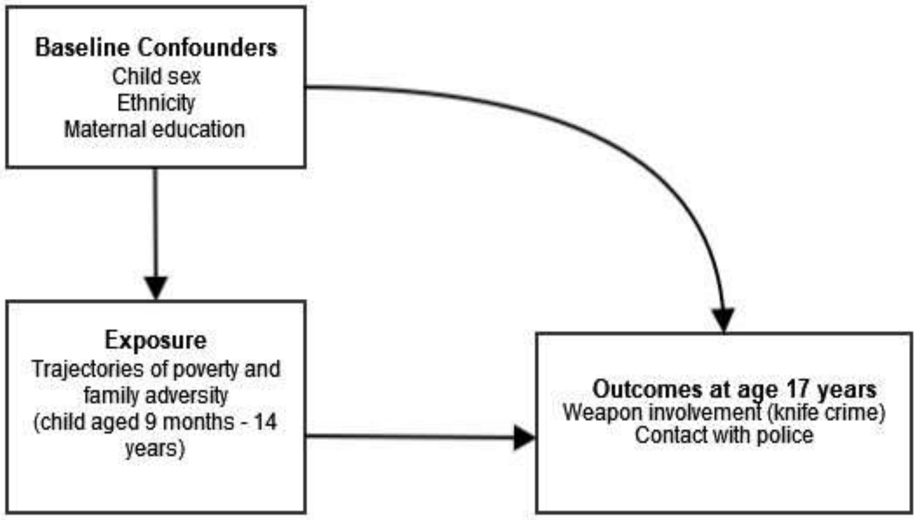

Supplementary Figure 2. Directed acyclic graph for the current study

Technical Appendix A. Population Attributable Fraction (PAF)

We calculated the PAF for exposure to any trajectory compared to the low poverty and adversity trajectory group according to the general formula:

$$PAF = \sum_{i=0}^{n} P_i \left(\frac{RR_i - 1}{RR_i} \right)$$

where P_i represents the proportion of cases in each exposure (i.e., each trajectory group) and RR_i represents the adjusted RR of adverse outcomes (i.e., police contact and weapon involvement) for each trajectory group compared to the low poverty and adversity group.

Supplementary Table 1. Population attributable fractions (**adjusted**) showing the proportional reduction in adverse outcomes that could be achieved if all children had the same exposure as those in the low poverty and adversity trajectory.

	**Police contact**	**Weapon involvement**
Persistent parental alcohol use	0.012 (-0.005-0.030)	0.018 (-0.023-0.058)
Persistent domestic violence and abuse	0.018 (0.004-0.031)	0.019 (-0.010-0.049)
Persistent poor parental mental health	0.033 (0.009-0.057)	0.055 (-0.006-0.113)
Persistent poverty	0.089 (0.047-0.129)	0.131 (0.009-0.237)
Persistent poverty and poor parental mental health	0.068 (0.039-0.095)	0.055 (-0.013-0.118)
	**0.220 (0.133-0.272)**	**0.278 (0.060-0.432)**

Note: The reference group is the low poverty and adversity trajectory. The PAF is calculated comparing two scenarios: scenario 1 (a hypothetical scenario in which all children were in the low poverty and adversity trajectory) with scenario 0 (the real world in which there are children in the low poverty and adversity and other trajectories).

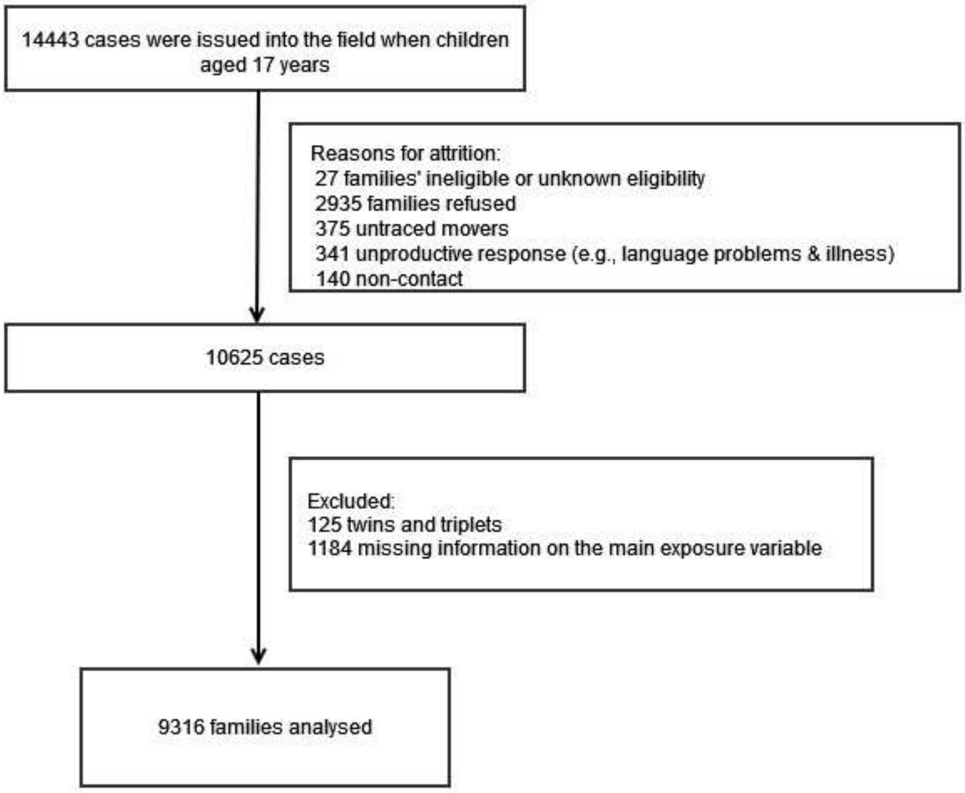

Supplementary Figure 3. Study flow diagram showing inclusion and exclusion of cohort participant

Supplementary Table 2. Associations of predicted poverty and family adversity trajectories and outcomes at age 17 years in the UK Millennium cohort study (unadjusted model)

	Outcomes	
**Variables**	**Police contact**	**Weapon involvement**
**Trajectories**		
Low poverty and adversity	Ref.	Ref.
Persistent parental alcohol use	1.11 (0.86-1.44)	1.24 (0.75-2.03)
Persistent domestic violence and abuse	1.53 (1.01-2.17)	1.84 (0.91-3.74)
Persistent poor parental mental health	1.24 (0.99-1.55)	1.77 (1.15-2.73)
Persistent poverty	1.67 (1.38-2.02)	2.12 (1.45-3.10)
Persistent poverty and poor parental mental health	2.20 (1.75-2.77)	2.52 (1.64-3.87)

Supplementary Table 3. Associations of predicted poverty and family adversity trajectories and outcomes at age 17 years in the UK Millennium cohort study, complete case analysis

**OR**	**Model***	Low poverty and adversity	Persistent alcohol use	Persistent domestic violence and abuse	Persistent poor parental mental health	Persistent poverty	Persistent poverty and poor parental mental health
Police contact	1	Ref.	1.20 (0.92-1.55)	1.77 (1.24-2.54)	1.27 (1.00-1.62)	1.64 (1.34-2.01)	2.42 (1.86-3.15)
	2	Ref.	1.16 (0.89-1.52)	1.75 (1.20-2.53)	1.31 (1.02-1.677)	1.68 (1.33-2.12)	2.34 (1.72-3.18)
	3	Ref.	1.10 (0.84-1.44)	1.50 (1.05-2.15)	1.28 (1.02-1.58)	1.65 (1.31-2.05)	2.10 (1.50-2.64)
Weapon involvement	1	Ref.	1.34 (0.79-2.28)	2.08 (1.04-4.14)	1.99 (1.28-3.10)	2.14 (1.45-23.15)	2.65 (1.64-4.30)
	2	Ref.	1.34 (0.78-2.29)	2.01 (0.98-4.10)	1.74 (1.09-2.77)	1.99 (1.23-3.22)	2.19 (1.21-3.97)
	3	Ref.	1.26 (0.76-2.09)	1.79 (0.86-3.67)	1.68 (1.06-2.66)	2.09 (1.30-3.36)	2.17 (1.30-3.62)

Note: * Model 1- crude model; Model 2 – adjusted for child's sex, maternal education and maternal ethnicity; Model 3 – adjusted for child's sex, maternal education, partner's education, maternal ethnicity, partner's ethnicity and child's ethnicity

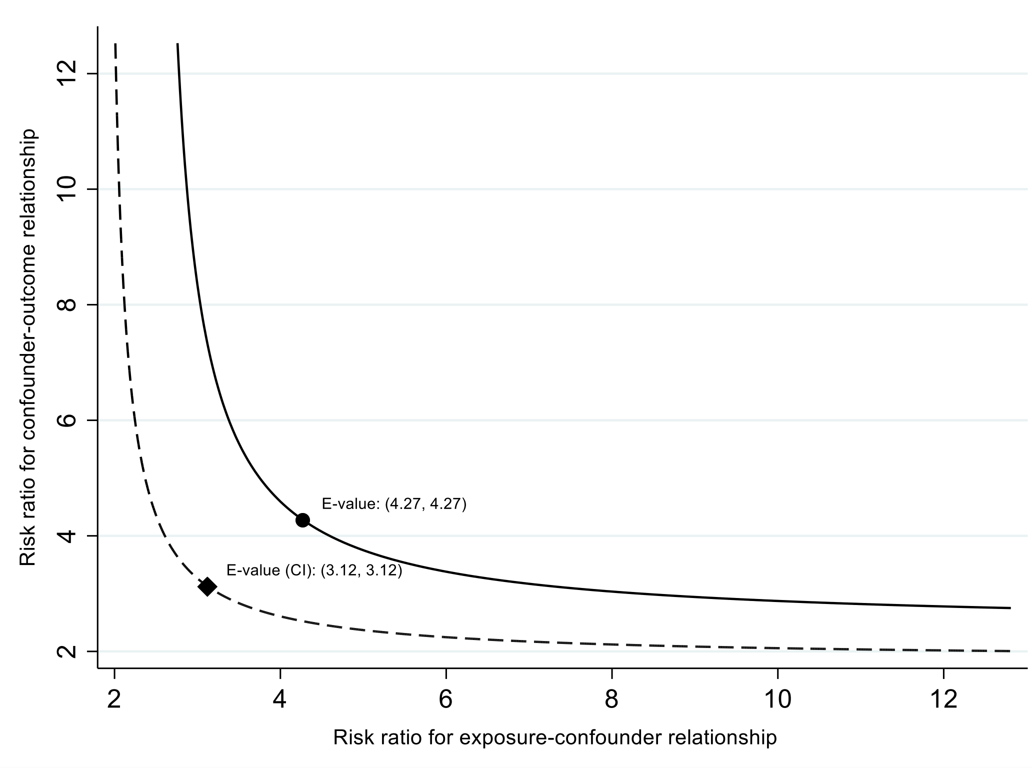

Supplementary Figure 4a. Sensitivity analysis for unmeasured confounding using E-values (police contact)

The E-value is 4.27 with a lower confidence limit (LCL) of 3.12. This means the unmeasured confounder would have to be associated with both family adversity (i.e., poverty and poor parental mental health) and police contact at age 17 by a risk ratio of 4.3 times each. To move the LCL to include no effect, association of an unmeasured confounder would need to be 3.1 or above.

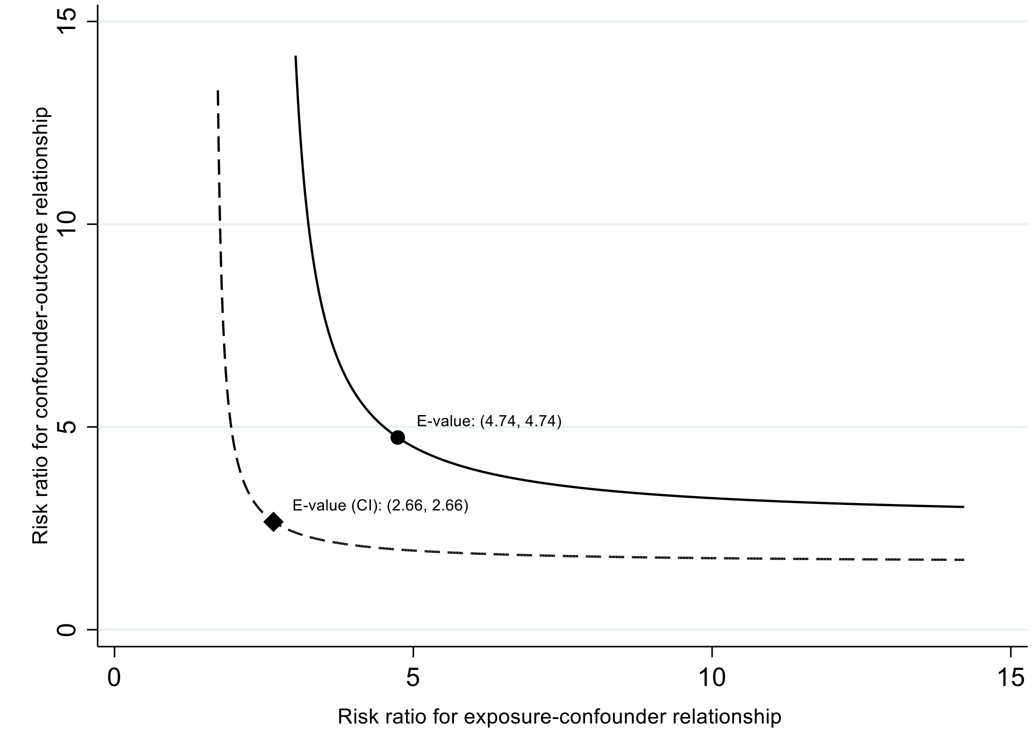

Supplementary Figure 4b. Sensitivity analysis for unmeasured confounding using E-values (weapon involvement)

The E-value is 4.74 with a lower confidence limit (LCL) of 2.66. This means the unmeasured confounder would have to be associated with both family adversity (i.e., poverty and poor parental mental health) and police contact at age 17 by a risk ratio of 4.7 times each. To move the LCL to include no effect, association of an unmeasured confounder would need to be 2.7 or above.

References

1. Adjei NK, Schlüter DK, Straatmann VS, et al. Impact of poverty and family adversity on adolescent health: a multi-trajectory analysis using the UK Millennium Cohort Study. *The Lancet Regional Health–Europe* 2022; **13**.
